# The role of mesenchymal stem cell transplantation for ischemic stroke and recent research developments

**DOI:** 10.3389/fneur.2022.1000777

**Published:** 2022-11-16

**Authors:** Li Zhou, Jiani Wang, Jiagui Huang, Xiaosong Song, Youlin Wu, Xia Chen, Yongjun Tan, Qin Yang

**Affiliations:** Department of Neurology, The First Affiliated Hospital of Chongqing Medical University, Chongqing, China

**Keywords:** mesenchymal stem cells (MeSH ID: D059630), stem cell transplantation (HSCT), ischemic stroke (IS), stem-cell based therapy, experimental treatment

## Abstract

Ischemic stroke is a common cerebrovascular disease that seriously affects human health. However, most patients do not practice self-care and cannot rely on the current clinical treatment for guaranteed functional recovery. Stem cell transplantation is an emerging treatment studied in various central nervous system diseases. More importantly, animal studies show that transplantation of mesenchymal stem cells (MSCs) can alleviate neurological deficits and bring hope to patients suffering from ischemic stroke. This paper reviews the biological characteristics of MSCs and discusses the mechanism and progression of MSC transplantation to provide new therapeutic directions for ischemic stroke.

## Introduction

Stroke is a common neurological disease affecting human survival and health; it is characterized by high morbidity, mortality, disability, and a high recurrence rate (1). Statistically, more than 13.7 million people suffer from strokes worldwide annually, and 5.8 million die (2). More remarkable, ischemic stroke incidence is increasing yearly due to the aging population and other reasons. Therefore, ischemic stroke has received increasing attention as the most common type (accounting for ~70% of strokes) (3).

Ischemic stroke is a pathological process caused by a blood circulatory disorder in the brain that leads to neuronal cell death or softening of the brain tissue. As a terminally differentiated cell, the death of a large number of neuronal cells leads to irreversible damage to brain tissue. Early recovery of blood volume in the ischemic area and reduction of nerve cell death are the key points in the treatment of ischemic stroke. However, treatments such as thrombolysis and mechanical thrombectomy benefit only 5% of patients because of narrow therapeutic windows and severe treatment complications (4–6). Thus, further research for safer and more effective ways is still warranted (7).

Stem cells are primitive and unspecialized cells that can develop into diverse specialized cells through mitosis and differentiation and have the potential to regenerate a variety of tissues and organs (8). Extensive preclinical evidence suggests that stem cell transplantation therapy can alleviate brain tissue damage by directional proliferation and differentiation of nerve cells and other pathways. A large number of abnormal nerve cell deaths can occur after an ischemic stroke, and stem cell transplantation will be a viable treatment to relieve neurological deficits in the future (9).

Various types of stem cells have been studied in animal models or clinical studies, such as neural progenitor cells (NPC), mesenchymal stem cells (MSC), endothelial progenitor cells (EPC), and human umbilical cord blood cells (HUCBCs) (10). However, these kinds of stem cells all have limitations in therapeutic effects. For example, EPC therapy faces ethical problems (11). NPCs are tricky to harvest and have a low proliferation rate (12). The treatment of engineered cells, such as induced pluripotent stem cells (iPSC), NT2N cells, CTX0E3, and SB623, is hampered by technology (13, 14). Nevertheless, it is worth noting that MSC cells have become the preferred cells for treating ischemic stroke due to their characteristics, such as high availability, efficient isolating and culturing, high immune tolerance, and fewer treatment complications. Furthermore, MSC cell therapy is not contrary to social ethics (15, 16).

In this paper, we analyze the biological characteristics of MSCs and the neuroprotective mechanism in treating ischemic stroke with the hope of providing new therapeutic directions for ischemic stroke.

## Overview of MSCs

MSCs were first described as spindle-bone marrow stromal cells adhered to plastic by Friedenstein and his colleagues in 1970 (17). Four years later, they found that MSCs can form colonies outside the body that adhere to the wall like fibroblasts. Hence, MSCs are also known as cluster unit fibroblasts (CFU-Fs) (18). In 1991, Caplan coined the term “mesenchymal stem cells” and predicted that these mesodermally derived cells would represent the main arsenal of autologous therapies for regenerative purposes (19). With their development in recent decades, MSCs have become the most widely studied stem cell population. They are widely used in clinical trials and/or the treatment of various diseases, especially neurological diseases (20, 21).

MSCs were isolated from bone marrow for the first time. MSCs have previously been isolated from a variety of tissues, such as the lung, liver, kidney, placenta, fallopian tubes, endometrial polyps, adipose tissue, dental pulp, salivary glands, inferior turbinate, umbilical cord blood, menstrual blood, and other tissues (22, 23). They are plastic-adherent and can express mesenchymal markers, including CD90, CD105, CD73, and others, but cannot express CD11b, CD14, CD19, CD34, CD45, and human leukocyte antigen (HLA)-DR (24). MSCs can be harvested from different tissues, and various donor characteristics restrict the surface markers, quality, and isolated numbers of MSCs. Currently, the most frequently reported sources of MSCs utilized in clinical trials are the bone marrow, adipose tissue, and umbilical cord. MSCs obtained from adipose tissue (AD-MSCs) can express CD49d and produce more HGF and VEGF than bone marrow-derived stem cells (BM-MSCs) (25). Compared with bone marrow-derived stem cells, the number of cells obtained from 1 g of fat tissue may be 500 times greater than that of the same weight of bone marrow (26). However, BM-MSCs are safer than AD-MSCs because they can promote the proliferation of existing cancer cells, especially breast cancer (27). Both BM-MSCs and AD-MSCs have significant neurotrophic potential to stimulate neurite growth in DRG-neurons despite different growth factors, which further supports the feasibility of MSC-based stroke treatment (28). Recent investigations into the transplantation of human umbilical cord mesenchymal stem cells (hUC-MSCs)in stroke models have displayed favorable results, including a reduction in infarct size, improved functional recovery, and increased expression of several neuroprotective factors (including VEGF and BDNF) (29). Yet, their isolation can be difficult (30). MSCs from other sources, such as canine-derived MSCs (cMSCs), have not obtained sufficient clinical evidence and cannot be directly applied (31) (Table 1).

**Table 1:** Advantages and disadvantages of MSCs from different sources.

**Type**	**BM-MSCs**	**AD-MSCs**	**UCB-MSCs**
Advantages	1. Powerful immunomodulatory properties; 2. More extensive clinical application than other stem cells (14).	1. Adequate organizational sources; 2. Stable growth and proliferation kinetics; 3. Pro-angiogenesis (produce more HGF and VEGF) (32).	1. Easily and safely collected by UCB banks (a non-invasive collection procedure with low immunogenicity); 2. High proliferative rates, enhanced stem cell capacity, and delayed senescence (33); 3. Maintain multipotency for longer periods (34).
Limitations	1. The extraction process is often accompanied by pain and other adverse reactions; 2. Marrow is quite low and decreases gradually with age (35).	1. It can promote the proliferation of existing cancer cells, especially breast cancer (36).	1. Difficult to isolate (37); 2. Efficacy may be limited by the route of administration (38).
The main clinical application	Organ transplantation; Hematologic malignancies; Graft-vs.-host-disease (GVHD) (39).	Auto-immune diseases (such as systemic lupus erythematosus, systemic sclerosis, scleroderma, and Crohn's disease) (39).	Hematopoietic diseases (39).
Commonalities	1. Can adherence to plastic; 2. Can express different markers, including CD90, CD105, CD73, and others, but not CD11b, CD14, CD19, CD34, CD45, and HLA-DR; 3. Multipotent differentiation potential (40); 4. Have a variety of mechanisms to rescue the damaged tissues.		

BMSCs, bone marrow-derived MSCs; ADMSCs, adipose tissue-derived MSCs; UC-MSCs: umbilical cord-derived mesenchymal stem cells; HGF, hepatocyte growth factor; VEGF, vascular endothelial growth factor; and CD, cluster of differentiation.

**Figure 1:**
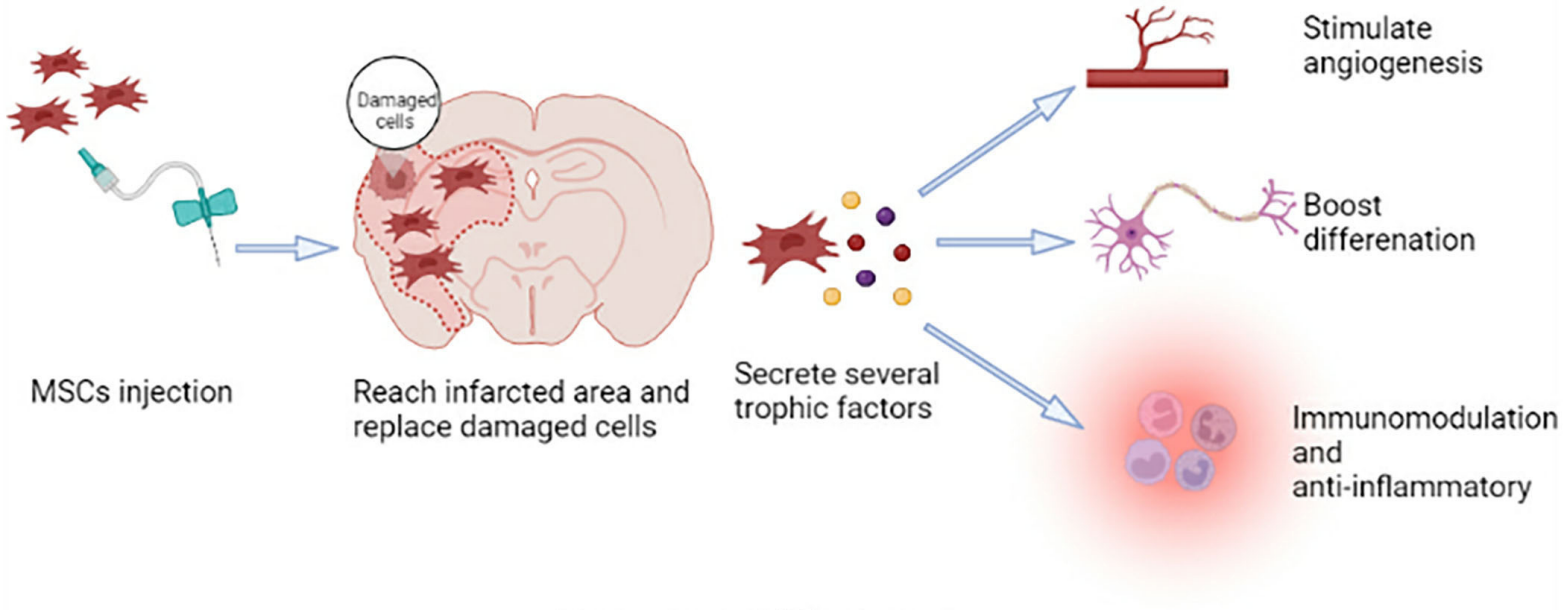
Mechanism of MSC therapy.

MSCs can self-renew and show polymorphic differentiation (41). They can differentiate into mesoderm cells (described above), endoderm (smooth muscle cells), and ectoderm (neurons) cells under certain conditions (42), which can promote the repair of various damaged tissues (41). Neural regeneration, including neurogenesis, angiogenesis, and synaptic plasticity, is crucial for functional recovery after a stroke. Because MSCs have the characteristics of plasticity, multidirectional differentiation, immunomodulation, and anti-inflammatory, they have the potential to participate in brain regeneration, which can promote tissue repair after ischemic stroke (Figure 1) (43).

## The role and research progress of MSC transplantation in the treatment of ischemic stroke

Since Azizi et al. published the first report on the transplantation of human BM-MSCs into the rat brain in 1998, an increasing number of studies on treating neurological diseases by MSC transplantation have been conducted. Moreover, MSC transplantation therapy is gradually shifting from laboratory to clinical therapy. Successful clinical studies demonstrate the clinical transformation of MSC transplantation in treating ischemic stroke. Researchers have adopted various methods (intravenous, artery, and intrathecal injection) to administer MSCs to patients with ischemic stroke. They have focused on the safety, feasibility, and short-term effectiveness of MSCs in treating ischemic stroke. This section summarizes several clinical trials to explore the feasibility of MSCs in treating patients with ischemic stroke.

### Plentiful preclinical studies of MSC transplantation therapy in ischemic stroke provide a theoretical basis for clinical practice

Medication, rehabilitation training, and physical union therapy have not been effective as experimental treatments for ischemic stroke. Except for thrombolysis and mechanical thrombectomy, no effective medications or procedures have yet been developed. In this situation, new therapeutic strategies using multiple mechanisms are sought, with MSC transplantation being one of them.

In the preclinical studies, researchers explore different sources of MSCs, feasibility, security, and specific mechanisms of MSC-based therapy. First, using *in vitro* models, they isolate MSCs from various tissues and demonstrate cell differentiation, neuroprotection, neurogenesis, and angiogenesis. In *in vivo* models, MSCs are injected into animals by different pathways. Researchers demonstrate that MSC transplantation has a potential therapeutic activity that can repair damaged brain tissue, and it seems feasible and secure (summarized in Table 2).

**Table 2:** Preclinical studies of MSCs for the treatment of ischemic stroke.

**Cell sources**	**Route**	**Time**	**Dosage**	**Outcome**	**References**
hATSCs	I.C	1-day after MCAO	1 × 10^6^ cells	Improved behavior; neural differentiation.	(44)
hMSCs	I.C	1-week after MCAO	75,000 cells	Improved behavior; neural differentiation.	(45)
HUMSCs	I.C	1-day after MCAO	5 × 10^5^ cells	Reduces the area of infarction, angiogenesis; improved behavior; neural differentiation.	(46, 47)
hMSCs	I.C	3-days after MCAO	5 × 10^5^ cells	Immune modulatory; endogenous neurogenesis.	(48)
rBMSCs	I.C	1-day after MCAO	1 × 10^5^ cells	Neurological function improved; reduced infarct area; decreased amount of apoptosis	(49)
hBMSCs	I.V	60 days post-stroke,	4 × 10^6^ cells	Reduce infarct area; improve systemic inflammatory response;	(50)
eMSCs	I.V	1-day after MCAO	20 × 10^6^ cells	Improved behavior; neural and endothelial cell differentiation; reduced infarct area.	(51)
B10 -hMSCs	I.V	1-day after MCAO	3 × 10^6^ cells	Improved behavior; neural cell differentiation; reduced infarct area; neurotrophic factors and cytokines produced.	(52)
HUMSCs	I.V	2-day after MCAO	Low-dose (1 × 10^4^ cells) and high-dose (1 × 10^5^ cells)	Immunomodulation; improved behavior; high dose (1 × 10^5^) of UC-MSCs improved functional outcomes.	(53)
rBMSCs	I.V	1 h after dMCAO	1 × 10^6^ cells	Immunomodulation; produce neurotrophic factors and cytokines.	(54)
rBMSCs	I.A	1-day after MCAO	2 × 10^6^ cells	Improved behavior; axon remodeling; angiogenesis.	(55)
rBMSCs	I.A	1, 6, 24, 48 h after MCAO	1 × 10^6^ cells	Improved behavior; reduced infarct area; 24 h after MCAO may be optimal timing for stroke.	(56)
hBMSCs	I.A	1-day after MCAO	1 × 10^6^ cells	Improved behavior; reduced infarct area; Methylation of ANP and BDNF promoter further decreased, which showed a significant increase in ANP and BDNF expression.	(57)
hBMSCs	I.A	1, 4, or 7 days after MCAO	1 × 10^6^ cells	Neuroprotection regulates reactive astrocytes and angiogenesis; these effects are timing-dependent.	(58)
Autologous ADMSCs	I.A	Three days after MCAO	2 × 10^6^ cells	Improved behavior; attenuated astroglial reactivity; inhibited cell apoptosis and promoted cellular proliferation	(59)
rBMSCs	Intranasal	Three days after MCAO	1 × 10^5^ cells	Improved behavior; reduced infarct area; HP-rBMSCs optimize the therapeutic efficacy	(60)
rBMSC	Intranasal	Three days after MCAO	1 × 10^6^ cells	Reduce infarct area; reduces motor deficits; endogenous neurogenesis;	(61)
rBMSCs	Intranasal	6 h (first), and 3 days (second) after neonatal Stroke model	1 × 10^6^ cells	Improved behavior; reduced infarct area; angiogenesis and neurogenesis.	(62)

I.C, intracranial administration; I.V, intravenous infusion; I.A, artery infusion; hATSCs, human adipose tissue stromal cells; rBMSCs, rat BMSCs; HP-rBMSCs, hypoxic pretreatment rBMSC; HUMSCs, human umbilical mesenchymal stem cells; BAO, basilar artery occlusion; and hBMSCs, human bone marrow stromal cells.

#### Model *in vitro*

The *in vitro* propagation of MSCs is a three-step process: Extracted from various tissues, MSCs are separated and obtained using density gradient centrifugation digesting culture before being cultured in a plastic cell tissue culture bottle for 3–5 days for further expansion. These steps are then repeated to expand adhered MSCs (63). The following are detailed procedures: To begin, isolate MSCs from multiple tissues such as bone, adipose tissue, tooth tissue, and others using Percoll or Ficoll density gradient centrifugation. Second, rinse MSCs with buffer once to eliminate contaminants before cultivation in 10% fetal bovine serum (FBS) or FBS substitutes, and incubate them in flasks at 37 °C in a humidified 5% CO2 incubator for 2 days. Non-adherent cells are removed by replacing the medium with a fresh one. Subsequently, the attached MSCs proliferate for 2–3 weeks with regular medium change. When the cells have grown to cover about 80% of the flask, it is critical to separate them and allow them to proliferate continually (63).

At present, researchers agree on the multi-lineage differentiation and transplantation potential of MSCs to replace lost tissue after ischemic stroke. When these isolated MSCs are treated with corresponding growth factors or induction medium, they can differentiate into various cell types from different blastoderms (64, 65). These differentiated neuron cells also have functional activity. For example, electrophysiological measurements show that the differentiated cells have voltage-gated sodium and potassium currents, which can be reversibly blocked by tetrodotoxin and tetraethylammonium, respectively (6, 9). However, we need more evidence to prove whether the differentiated cells can fire repetitive action potentials (64).

In addition, MSCs have great neuroprotective effects and promote neurite outgrowth *in vitro*. Liu et al. claim that BM-MSCs can promote the survival of oxygen-glucose deprivation (OGD) injured neurons, promote axonal outgrowth, and upregulate the expression of GAP-43 when they are cocultured for 48 h with neurons following OGD injury (66). BM-MSCs can also stimulate neurite outgrowth of DRG neurons (67). When hippocampal slices or cortical neurons are cocultured with hMSCs or MSC-derived SB623 cells separated by a semi-porous membrane or with MSC- or SB623 cell-conditioned medium following OGD, neural cell death or damage is decreased. Moreover, 11 neurotrophic factors are identified as secreted by MSCs and/or SB623 cells, and most of them are potentially beneficial to neural tissue following an ischemic insult (68). Furthermore, BM-MSCs from normal healthy and cerebral ischemia rats increase neuronal survival and connectivity in glial-neuron mixed cultures (69). These reports support the fact that MSCs have neuroprotective effects and stimulate neurite development *in vitro*.

Lastly, the ability of MSCs to stimulate angiogenesis, participate in vascularization, and re-establish a blood supply is evaluated in *in vitro* models. It is also the fundamental process of tissue repair (70). In *in vitro* models, MSCs can differentiate into endothelial lineage cells to protect ECs against hypoxia-induced cell death (71), promote the formation of endothelial rings (65), improve the paracrine activity of angiogenic growth factors and EC migration, and form mature vascular tissue (70). Hypoxic preconditioning enhances the pro-angiogenic effects of MSCs by increasing the expression of angiogenic growth factors and boosting the proliferation and migration of ECs (72).

Thus, MSCs show great promise *in vitro*, including the potential for cell differentiation, neuroprotection, neurogenesis, and pro-angiogenesis. Therefore, several studies have ulteriorly transplanted MSCs into animal models of ischemic stroke and evaluated the outcomes as discussed below.

#### Model *in vivo*

In *in vivo* studies, researchers focus more on the effectiveness, including the neuroprotection, immune regulation, angiogenesis, neurogenesis, and other potential of MSC transplantation, as well as the feasibility and security. The commonly used transplantation pathways are stereotactic administration (intrastriatum and intraventricular), systemic administration (intra-vein IV, intra-arterial IA), and other routes used, such as the intranasal route (73). Peripheral transplantation may be more appropriate for acute stroke patients because the BBB is permeable in this period, and stem cells are easy to pass through, whereas direct stereotaxic cell administration seems appropriate for fixed, chronic stroke patients (74).

Stereotactic transplantation (intracerebral and intraventricular), also called intracranial transplantation, is used to transplant MSCs into various brain parts. In particular, MSCs can be precisely administered to a selected location by intracerebral (IC), and intraventricular (IV) techniques, which are the earliest transplantation means used in experiments. Moreover, more MSCs can reach the target sites of brain injury in this way without going through the whole body's metabolic cycle (75). Zhao et al. pioneer intracranial transplantation of MSCs and find that MSCs migrate toward the infarct region of the brain and can survive in the host brain and promote functional recovery (45). Transplanted hMSCs can differentiate into mature neurons and gliacytes, which are observed at the grafting site and along the migration pathways (47). Rats are given two injections of grafted HMSCs into the infarct cortex within 24 h after MCAO, which is shown to substantially enhance neuronal metabolic activity, facilitate repair of the infarct cortex, and improve functional outcomes in rats (46). By expressing neuroprotective and growth-associated cytokines, intracerebral transplanted MSCs also increase neuronal activity, decrease cell death, and promote angiogenesis in the infarct cortex (47). They also modulate the immune response and produce TGF-, which inhibits MCP-1 secretion and restricts the infiltration of CD68 + cells into the damaged tissue (48). When MSCs are injected into the ventricles and cisterns, they are distributed throughout the brain and spinal cord with cerebrospinal fluid. It can effectively improve the survival rate of transplanted MSCs without going through general metabolism. It is proven that hADSCs can migrate safely into damaged areas and survive when injected into subarachnoid space through the superior orbital fissure (76). However, it may have higher risks due to the invasive procedures and complexity of stereotaxic procedures, which are unbearable for most clinical patients (54).

Systemic administration (Intra-vein IV, Intra-arterial IA): To overcome the invasiveness and complexity of stereotaxic transplantation, researchers find that a microtrauma method can be achieved. Bone marrow MSCs injected intravenously or arterially can migrate into the infarct area to promote neurogenesis and angiogenesis (77), facilitate axonal sprouting and remyelination (55), attenuate microglia/macrophage infiltration (69, 78), inhibit gliosis and apoptosis in the ischemic brain, and reduce infarct volume and improve functional outcomes in ischemic stroke rats (59). The intravenous route has advantages such as simplicity, safety, high feasibility, repeatable operation, high patient acceptance, and small adverse reactions. Furthermore, the IV MSCs will preferentially migrate to the spleen (50) and abrogate the systematic inflammation-plagued secondary cell death. However, IV delivery has the “first-pass effect” after transplantation; that is, the transplanted cells are distributed in the liver, lung, spleen, kidney, bone marrow, thymus, and even skin and tumors on the way. Thus, only a few transplanted cells reach the lesion site (77). Therefore, IV delivery needs a large number of cells to be injected into patients, which may increase potential side effects and the cost of the treatment (79). As the number of IA catheter-based interventions depending on stroke therapy characteristics (such as mechanical thrombectomy and catheter-directed thrombolysis for patients with penumbra) is constantly increasing, it seems that IA cell injection is ideally suited in the specific setting (80, 81). The arterial route has a high transplantation rate, does not pass through other organs, reaches the cerebral cortex and peripheries of the lesion directly and quickly, expresses glial and neuronal markers, and induces faster improvement of neurological function in animals (56). However, it may form microemboli and cause new infarctions. Intra-arterial cell administration at low doses may reduce the risk of embolic complications and promote functional recovery. However, more research is still needed to determine the most effective dosage (82).

Another route: Intranasal administration is a simple, convenient, and non-invasive delivery method that bypasses the BBB, directly guides therapeutics to the CNS (83). reaches the frontal part of the brain within 30 mins after administration, and distributes throughout the whole brain after 3 h (84). Wei et al. first provided information on intranasal cell delivery for treating ischemic stroke (60). In the study, hypoxic-preconditioned BM-MSCs were intranasally delivered 24 h after stroke. The result shows that BM-MSCs reach the ischemic cortex and deposit outside vasculatures as early as one and a half hours after administration, upregulating expressions of MMP-2, MMP-9, and the SDF-1 receptor CXCR4, reducing cell death and infarct volume and improving functional outcomes (60). This method is effective and minimally invasive and has been used in the treatment of neonatal stroke. In the neonatal HI model, intranasal delivery of MSC- and MSC-BDNF significantly reduces infarct size and gray matter loss, increases Ki67-positive cell number in the SVZ, enhances endogenous repair processes, and effectively reduces long-term functional impairment (61). In the perinatal brain injury (PBI) model, MSC-exosomes reduce gray and white matter injuries and improve functional recovery (84).

Additionally, intranasally administered BM-MSCs improve local cerebral blood flow in the ischemic cortex after a neonatal stroke and significantly decrease infarct size and BBB disruption. They also promote angiogenesis and neurogenesis (62). These reports indicate that intranasal-delivered BM-MSCs are feasible and effective, but more research and exploration are still needed.

In order to make cell therapy more viable, it is necessary to clarify cell migration, viability, and efficient delivery to target locations after transplantation. In a study by Archana Mukherjee, the researcher labeled umbilical cord-derived MSCs with ^51^Cr (85). After 96 h of being injected into healthy Swiss mice *via* the tail vein, retention of activity in the blood and high uptake of ^51^Cr in the kidneys were still observed. The rate and proportion of cells reaching the damaged site vary with administration. Factors determining cell distribution have not been fully elucidated. A study by Ilya et al. demonstrated a high coefficient of determination of up to 30% correlation between the distribution of IA transplanted MSCs and brain perfusion (86). The apoptosis of MSCs is the same as normal cells, which are cleared *via* the hepatobiliary and renal routes with time. Biodistribution and imaging studies are desired in animal models to disclose more mechanisms. A study by Seungho Lim and colleagues provides dual-modal stem cell imaging probes as a new method of labeling human AD-MSCs to realize non-invasive and precise tracking after transplantation in living subjects, which will be a forceful tool in the future (87).

### Increasingly successful clinical studies provide evidence for the possibility of clinical transformation of MSC transplantation in the treatment of ischemic stroke

In recent years, clinical trials have focused on the safety, feasibility, and short-term effectiveness of MSCs in treating ischemic stroke (9) (summarized in Table 3). Transplantations of MSCs in human patients began in 1995, with most early trials focusing on the potential benefits of autologous MSCs in promoting the engraftment of hematopoietic stem cells in the setting of hematological malignancy (105). In 2005, the first randomized case-control clinical trial was conducted to evaluate the effects of intravenous autologous MSCs on patients with middle cerebral artery infarction (106). Thirty patients from 30 to 75 years (*n* = 5 in the MSCs group and n = 25 in the control group) were randomized to receive 1 × 10^8^ BM-MSCs intravenously within 7 days of the stroke. It showed that BM-MSCs could improve functional recovery, and no adverse events were associated with transplantation during the 1-year follow-up period. Three cases of ischemic stroke and one case of hemorrhagic stroke in the territory of the middle cerebral artery were treated with 2 × 10^7^ umbilical cord-derived MSCs by a catheter to a near lesion site in 2012 by Jiang et al. (88), and the patients were followed up for 6 months after the procedure. Muscle strength was improved in two ischemic stroke patients, and there were no adverse events. Subsequently, in 2014, 8 patients with large cerebral infarction or anterior arterial infarction were treated with MSCs alone (intravenous injection of MSCs at 0.5 × 10^6^/kg body weight four times) or MSCs combined with neural stem/progenitor cells (NSPCs) (intravenous injection of MSCs at 0.5 × 10^6^/kg body weight the first time, and then injected three times at 5 × 10^6^/patient MSCs and 6 × 10^6^/patient neural NSPCs) through the cerebellar bulbar cistern. Except for the occurrence of slight dizziness and low fever within 2–24 h, there were no other serious adverse reactions, and levels of disability and daily living ability improved. The result indicates that the combined transplantation of NSPCs and MSCs is a safe and feasible method to improve neural function (93). In 2018, Deng et al. (96) included 108 patients with IS within 30 to 90 days of onset and were randomized into an experimental group (*n* = 59) and a control group (*n* = 59). Then allogeneic BM-MSCs (1 × 10^6^ cells/kg body weight) were injected intrathecally four times a week. All patients underwent a detailed functional assessment and magnetic resonance imaging before the cell infusion and at 1-year intervals, then assess its safety and feasibility. Currently, it is in the phase II experiment. In 2020, a single-center, open-label, randomized controlled trial enrolled patients aged 18–70 following moderate-severe ischemic carotid stroke (National Institutes of Health Stroke Scale, NIHSS>10) for < 2 weeks was conducted. Patients were randomized 2:1 to receive intravenous 1–3 × 10^8^ BM-MSCs (*n* = 31) or not (*n* = 16), with a 2-year follow-up. Researchers found that the intravenous injection of autologous MSCs was safe and feasible for treating moderate to severe stroke (100). In 2021, Chung et al. confirmed that intravenous application of preconditioned autologous MSCs with autologous serum was feasible and safe for patients with chronic major stroke (101). A neuroimaging study also showed positive changes in network reorganization to facilitate motor recovery after stroke (102). A phase 2, single-center, assessor-blinded randomized controlled study by Zhe Kang Law and colleagues estimated the safety, tolerability, and efficacy of intravenous infusion of autologous MSCs (104). The treatment group (received culture-expanded autologous BM-MSCs intravenously) and the control group (received standard medical care) had recovery effects, but there was no significant difference between them. The 17 patients were all safe and welltolerated. Consistent with these results, intravenous injection of autologous MSC administration is safe and feasible for treating stroke. In 2022, a Phase I open-label study by Tsung-Lang Chiu adopted autologous adipose-derived stem cells to treat three chronic stroke patients by stereotactic implantation, which improved patients in many aspects without any adverse effects observed (91). Another study by Elena de Celis-Ruiz also demonstrated the safety of intravenous adipose tissue-derived mesenchymal stem cell therapy from the first 2 weeks after ischemic stroke to 24 months of follow-up (89, 91). A first-in-human, open-label intervention study by Lisanne M. Baak and team used intranasally delivered bone marrow-derived allogeneic MSCs for neonates with perinatal arterial ischaemic stroke (98). All ten enrolled neonates were welltolerated with the therapy, and no serious adverse events were observed until 3 months of age follow-up.

**Table 3:** Clinical studies of MSCs for the treatment of ischemic stroke for the past 10 years.

**Cell sources**	**Patient population; the MSCs/control group;**	**Route**	**Time**	**Dosage**	**Outcome**	**References**
UCMSCs	40–50 years old; Within 3 months of the stroke; Four male patients; No control group or blinding.	I.A	Within 3 months of the stroke	2 × 10^7^ cells.	Follow up 6 months; Improving muscle strength and increase of mBI score; No adverse events.	(88)
Allogeneic ADMSCs	60–80 years old; < 12 h of stroke; NIHSS of 8–20 scores 4/13	I.V	Within 2 weeks of the stroke	1 × 10^6^ cells/kg	Follow up 24 months; improvement of clinical scores of NIHSS; No adverse events. No tumor development	(89, 90)
Allogeneic ADMSCs	65–80 years old; NIHSS of 16–20 scores; Post-stroke between 6 months and ten years; 3/3	I.C	1 week after inclusion.	1 × 10^8^ cells	Follow up six months; improvement of clinical scores of NIHSS, Barthel Index, Berg balance scale, and F-M; No adverse events.	(91)
Allogeneic AD-MSCs	≥18 years old; NIHSS of 8–20 score (with at least 2 in sections 5 and 6) Treatment within four days (±1 day) from the onset 15/30	I.V	Within the first four days from stroke onset	1 × 10^6^ cells/kg	Results not released. (estimated end date is July 2023.)	(92)
HUMSCs AND hNSPCs	30–85 years old; Three females and three males; Acute/subacute and during stroke sequelae; Three patients had IV MSCs four times, and three had cotransplantations of MSCs and NSPCs four times.	I.V and I.C	Acute/subacute and during stroke sequelae.	The first group IV MSCs (0.5 × 106/kg) four times; the second group first IV MSCs (0.5 × 10^6^ /kg) followed three times by IC MSCs (5 × 10^6^/patient) and NSPCs (6 × 10^6^/patient) 2.5 × 10^6^, 5.0 × 10^6^, or 10 × 10^6^ cells.	Follow up two years; Safe and feasible; different degrees of clinical and functional improvement; No adverse events.	(93)
Modified BMSCs (SB623 Cells)	Mean age of 61 years old; NIHSS (SD): 9.44 score; Within 6 to 60 months of stroke; 11 females, seven males; 16/36.	I.C	The mean poststroke interval was 22 months.	2.5 × 10^6^, 5.0 × 10^6^, or 10 × 10^6^ cells.	Follow up two years; Significant improvement of clinical scores of ESS, NIHSS, and F-M; No adverse events.	(94, 95)
Allogenic BMSCs	18–75 years old; NIHSS of 15–25 score; 30 to 90 days; 59/59.	I.C	30 to 90 days.	Four times 1 × 10^6^ cells/kg.	Results not released.	(96)
Allogenic BMSCs	≥18 years old; NIHSS≥6 score; >6 months of stroke; 27 females, nine males; Phase 1 (*n* = 15), phase 2 (*n* = 21).	I.V	>6 months of stroke.	In phase 1 (*n* = 15), each dose (0.5, 1.0, and 1.5 × 10^6^ cells/kg body weight); phase 2 (*n* = 21) received 1.5 × 10^6^ cells/kg.	Follow up 1 year; Decreasing of NIHSS/Barthel Index score; No adverse events.	(97)
Allogenic BMSCs	Neonates born at full term (≥36 weeks of gestation); MRI-confirmed PAIS in the middle cerebral artery region; 10/10.	Intranasal	Within seven days of presenting signs of PAIS	One dose of 45–50 × 10^6^ cells.	Follow up 3 months; improvements on MRI; No adverse events. (except for a mild transient fever without the need for clinical intervention.)	(98)
Autologous BMSCs	12 patients (four females, eight males); NIHSS between 4–15; Three months to two years after stroke; 6/6.	I.V	21+7 days after inclusion.	5~6 × 10^7^ cells.	Follow up 1 year; Significant improvement of mBI score; No adverse events.	(99)
Autologous BMSCs	18–70 years old; NIHSS>10 scores; within 2 weeks of stroke; 11 females, 22 males; 16/15.	I.V	Three weeks after inclusion.	Low-dose MSCs (1 × 10^8^); high-dose MSCs (3 × 10^8^).	Follow up two years; Decreasing of NIHSS score; No adverse events.	(100)
Autologous BMSCs	30–75 years old; NIHSS of 6–21 score; Within 3 months of the stroke; 39/15.	I.V	After being included.	1 × 10^6^ cells/kg	Follow up two years; feasible and safe Have an improvement in leg motor functionality; No adverse events.	(101–103)
Autologous BMSCs	30–75 years old; NIHSS of 10–35 score; within two months of stroke; 9/17.	I.V	After being included.	2 million cells/kg	Follow up 1 year; improvement in median infarct volume; No adverse events.	(104)

m-NIHSS, motor NIHSS; ESS, European Stroke Scale; F-M, Fugl-Meyer; MRS, Modified Rankin Scale.

These clinical trials have tried all of the common modes of drug delivery, including stereotactic administration, intravenous injection, and intranasal routes, and patient ages range from newborn to middle-aged and elderly. The existing results provide confirmed data for the safety and feasibility of MSC therapy. However, given the differences in patient population, cell origins, time of administration, and drug delivery systems, as well as small sample sizes and lack of randomization and double-blind control, the interpretation of these results is limited. Therefore, higher quality randomized clinical trials, including better phenotypes and larger series, are still necessary to provide more reliable data to further clarify the safety and efficacy of MSC-based therapy in cerebrovascular disease.

## More new ideas for MSC-related therapies

SB623 cells are a gene-modified derivate of MSCs using gene transfection with Notch intracellular domain (NICD) and are subsequently followed by the administration of certain trophic factors (107). Though the transfected plasmid gets lost because of the expansion and split of cells, patterns of DNA methylation and protein expression have been altered. Compared to hMSCs from the same donors, preclinical studies showed that SB623 cells had higher secretion of angiogenin, angiopoietin-2, HB-EGF, and VEGF levels in the conditioned medium (108). SB623 cells also secrete higher-level factors that protect cells and enhance MSC migration after a hypoxic injury, such as DKK-1, IL-6, IL-8, MCP-1, and GM-CSF (68, 109). These trophic factors play a pivotal role in anti-inflammatory and immunosuppressive effects.

They are intended for use as an allogeneic cell therapy for chronic motor deficiency, particularly after a stable stroke, due to their ability to produce trophic factors, encouraging neuronal cells' reconstructive approach. In the completed 2-year phase 1/2a, open-label, a single-arm study by Gary K. Steinberg and his colleagues, 18 patients with stable, chronic stroke received SB623 cell implantation therapy (94). At 12 months after treatment, there were many meaningful developments in measures assessed for acute and long-term outcomes, like the mean scale scores of ESS, NIHSS, F-M total score, and F-M motor function total score. During the 2-year follow-up of treatment-emergent adverse events (TEAE), all patients experienced at least one TEAE, although no evidence suggested that it was probably related to SB623 cell treatment. All patients were generally safe and welltolerated at 2 years (94, 95).

Some researchers considered that combination cell therapy for MSCs might interact well with other types of stem cells. In a study by Seyed et al. BMSCs and neural stem cells (NSCs) were used together to treat MCAO rats (49). From a theory basis, MSCs could provide an appropriate microenvironment for NSCs after stroke *via* immunomodulation, and anti-inflammatory and NSCs have a great capacity to differentiate into neural lineage cells. Luckily, they got satisfactory results: In the group receiving combination therapy, neurological function was improved, infarct area was reduced, and they had the lowest amount of apoptosis. This therapy is effective in the prevention of strokes as well (110). A study published by Li-yan Qiao in 2004 also proved its effectiveness and safety (mentioned on page 11, line 294) (93). We believe these new therapies can lead to better post-stroke recovery for patients.

## Concluding remarks

The potential of MSCs in the treatment of ischemic stroke is huge. In this article, we review MSCs in IS therapy. Although a large number of preclinical and clinical studies support their safety and restorative effects, each patient with ischemic stroke has different symptoms and physiological conditions. Thus, many key issues must be resolved before the clinical application, like optimal cell source, dosage, transplantation time window and pathway, and adverse event monitoring and management. Therefore, a better understanding of the mechanism of stem cell treatment of stroke will be required to help solve the above problems. The data regarding their exact mechanisms of action remain incompletely clear. The paracrine effects of MSCs, a crucial part of their therapeutic potential, have powerful neurotrophic, neuroprotective, angiogenesis, and neurogenesis activity. However, exploring the interaction between various soluble cytokines in the ischemic brain is necessary. In addition, their distinctive immunological profile supports their clinical application, especially as a product with the use of allogeneic MSCs. Moreover, the cell replacement and modulating multicellular fate of MSCs have a significant impact on the repair of brain tissue and are worth further study in the future. In conclusion, it seems that MSCs can be utilized as therapeutic candidates in stroke therapy and may pave the way for new treatments in the near future to improve neurologic function, survival, and quality of life for patients with ischemic stroke.

## Author contributions

QY designed and approved the final manuscript for publication. LZ and JW wrote the manuscript. JH, XS, YW, XC, and YT collected the references and modified the manuscript. All authors contributed to the article and approved the submitted version.

## Funding

This study was supported by the National Natural Science Foundation of China (Grant No. 82171456 and 81971229, to QY).

## Conflict of interest

The authors declare that the research was conducted in the absence of any commercial or financial relationships that could be construed as a potential conflict of interest.

## Publisher's note

All claims expressed in this article are solely those of the authors and do not necessarily represent those of their affiliated organizations, or those of the publisher, the editors and the reviewers. Any product that may be evaluated in this article, or claim that may be made by its manufacturer, is not guaranteed or endorsed by the publisher.
